# Attachment and need to belong as moderators of the relationship between thwarted belongingness and suicidal ideation

**DOI:** 10.1186/s40359-023-01080-y

**Published:** 2023-02-20

**Authors:** Franziska Dienst, Thomas Forkmann, Dajana Schreiber, Inken Höller

**Affiliations:** Department of Clinical Psychology, University of Duisburg-Essen, Universitätsstraße 2, 45141 Essen, Germany

**Keywords:** IPTS, Thwarted belongingness, Need to belong, Attachment

## Abstract

**Background:**

In the Interpersonal Theory of Suicide, thwarted belongingness is presented as a main predictor for suicidal ideation. Studies only partially support this prediction. The aim of this study was to examine whether the heterogenous results are due to moderating effects of attachment and the need to belong on the association between thwarted belongingness and suicidal ideation.

**Methods:**

Four hundred forty-five participants (75% female) from a community sample aged 18 to 73 (*M* = 29.90, *SD* = 11.64) filled out online questionnaires about romantic attachment, their need to belong, thwarted belongingness, and suicidal ideation cross-sectionally. Correlations and moderated regression analyses were conducted.

**Results:**

The need to belong significantly moderated the relationship between thwarted belongingness and suicidal ideation and was associated with higher levels of anxious attachment and avoidant attachment. Both attachment dimensions were significant moderators of the relationship between thwarted belongingness and suicidal ideation.

**Conclusion:**

Anxious and avoidant attachment as well as a high need to belong are risk factors for suicidal ideation in people with thwarted belongingness. Therefore, attachment style and need to belong should both be considered in suicide risk assessment and therapy.

## Background

With about 700 000 deaths per year, suicide is one of the most common causes of death worldwide [1]. Even though many risk factors for suicidal ideation and behaviour have been identified, 25% of all psychologists experience a patient’s suicide in their career [2]. Different psychological models and theories have been postulated to understand and predict suicidality. While most theories consider suicidal ideation and behaviour as one unitary construct, the Interpersonal Theory of Suicide [3] describes the desire for death separately from the ability to engage in suicidal behaviour [4]. The theory postulates that suicidal ideation (SI) is developed when the interpersonal constructs perceived burdensomeness (PB) and thwarted belongingness (TB) occur at the same time. PB describes the perception of being a burden for someone, which is associated with risk factors like family conflicts, unemployment, and physical illness [4]. TB includes loneliness and a deficit of positive reciprocal relationships. The subjective perception of both factors as well as feelings of hopelessness and these states being stable and unchangeable are determining for the development of an active suicide desire [5]. Finally, the Interpersonal Theory of Suicide proposes that the capability for suicide, which occurs through habituation to self-harm or past suicide attempts, is necessary for the transition from the desire to die to actual suicidal behaviour [4]. While the prediction of SI by PB is confirmed in most studies across different samples, the empirical support for TB is ambiguous [6, 7]. Several studies failed to confirm a significant association of the development of SI and TB or reported smaller correlations for TB than for PB [6]. TB also explained significantly less variance in SI [7]. Prospectively, PB was the more robust predictor, and increased the probability of passive SI more than TB [8, 9].

Due to the heterogeneous findings on TB, other factors appear to play a role in the development of SI. One factor discussed here is the need to belong (NTB). According to Baumeister and Leary [10], the need to form and maintain lasting, positive, and meaningful interpersonal relationships is a basic human motivation. The need is satisfied by regular and pleasant social interaction that is based on mutual interest and affection. A frustrated NTB as a consequence of an inadequate belonging is associated with decreased well-being and physical health, negative affect, anxiety, and depression. Although all humans have an NTB, people differ in its form, intensity, and expression. People with a high NTB feel lonelier when they are rejected and experience more negative emotions than people with a low NTB [10, 11]. Despite mutual covariations with loneliness and reciprocal caring relationships, the NTB is distinct from TB. TB is a mental state arising when the desire for social connectedness is not adequately satisfied and can be experimentally induced [4]. In contrast to that, the NTB is a dispositional trait and interindividual differences are independent of perceived acceptance, support, or rejection [10]. An unmet NTB leads to the state of TB [5]. High belongingness needs are not generally associated with decreased well-being [12]. However, people with a high NTB are more affected by discontent in relationships [13]. This mismatch between a high NTB and low relationship satisfaction to has been shown to be a significant predictor of suicidal ideation and behaviour [12]. This suggests that frustrated belongingness needs may be a risk factor for SI, particularly in people with high NTB.

**Fig. 1:**
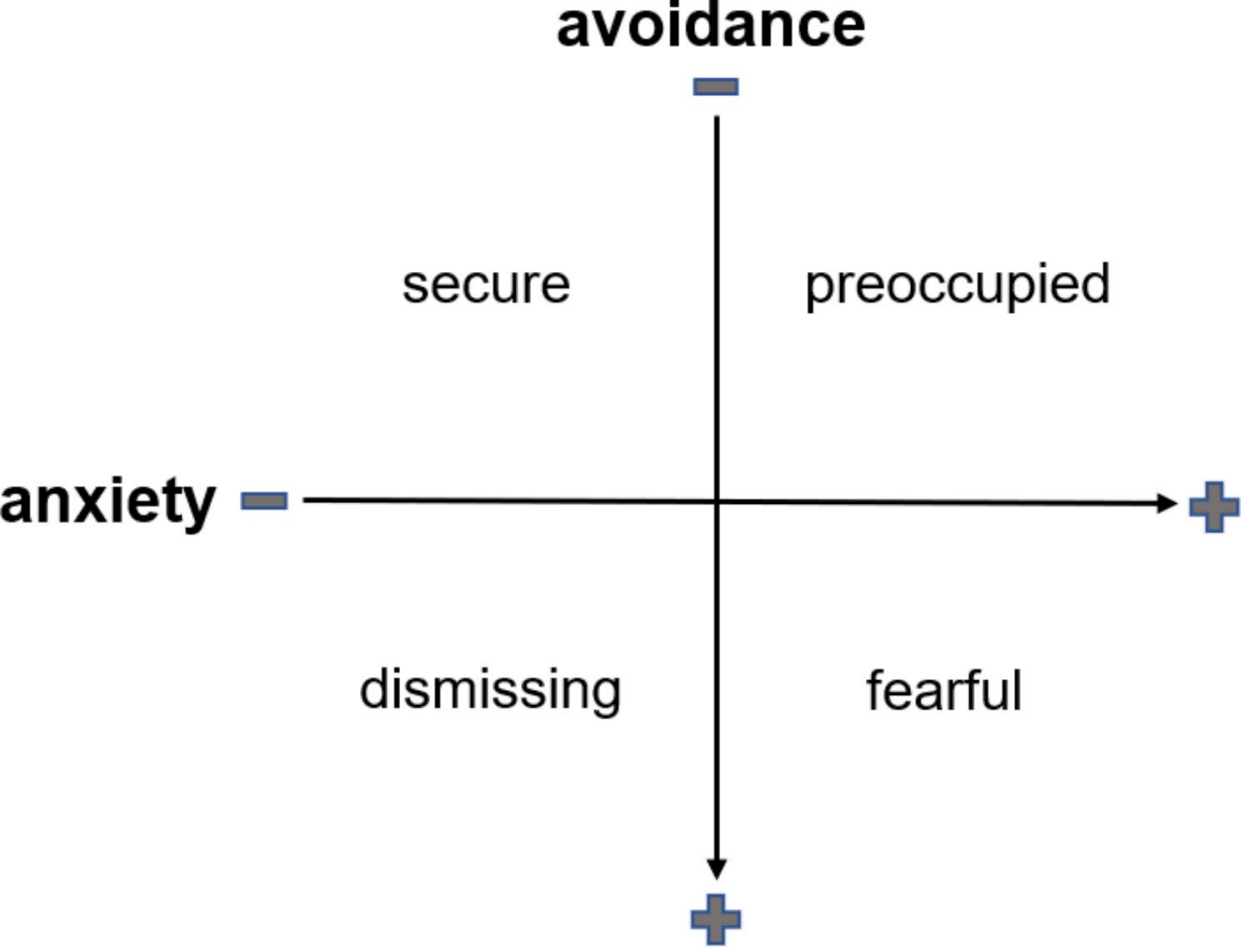
The four adult attachment styles depending on the dimensions anxious and avoidant attachment according to Neumann et al. [22]

The second factor discussed here is attachment security. Bowlby’s [14] attachment theory describes the development of close relationships between children and their primary attachment figure to ensure closeness to the caregiver [14, 15]. Attachment security is determined by the interaction quality with the attachment figure [16]. Ainsworth et al. [16] introduced three distinct patterns of attachment based on children’s former experiences. Secure, anxious-ambivalent, and anxious-avoidant attachment patterns differ in the reactions to the separation from and return of the caregiver (16). The responsiveness of the attachment figure is represented as an internal working model that influences relationships beyond the one with the own family later in life [17]. Attachment research focussed on parent-child-interactions for a long time, but Hazan and Shaver [18] applied the conceptualization of attachment on adult romantic relationships. The authors assumed that the internalized representations are consistent across adult attachment [18]. Bartholomew’s Four-Category Model of Attachment [19] describes distinct adult attachment styles determined by the underlying working model of the self and others. Secure attachment results from positive attachment models. A negative self-model is connected to high dependence on others and relationship dedication in a preoccupied attachment [20]. Negative experiences with others and frustrated attachment needs can result in either a fearful attachment style with a desire for belonging combined with fear of rejection or a dismissing attachment style, which is associated with the suppression of attachment needs and the fear of dependency [19, 20]. By now, a dimensional perspective on attachment is established in adult partnership research. Hence, attachment styles are understood as areas in a two-dimensional space with the dimensions avoidance of proximity and dependency, and anxiety of being abandoned [21]. Both dimensions have been confirmed by factor analyses and provide the basis for frequently used self-report questionnaires such as the Experience in Close Relationships (ECR; 21). Figure 1 shows the relations between Bartholomew’s [20] attachment styles and the current dimensional perspective on attachment. The dimensions anxious attachment and avoidant attachment were used in the present study.

Even though the attachment style is distinct from the strength of a person’s general NTB, there are conceptual similarities. This is supported by Leary et al. [11] who reported a moderate positive correlation between the NTB and anxious attachment. Additionally, several studies investigated associations of attachment with suicidality and TB. Adolescents with fearful and preoccupied attachment were significantly more likely to experience SI than those with dismissive or secure attachment styles [23]. Furthermore, anxiously attached adolescents were overrepresented in a case group with a history of suicidal ideation and behaviour [24] and a high-suicide risk group [25]. Patients with higher adult attachment anxiety were also more likely to report a suicide attempt history than patients with low attachment anxiety [26]. In a sample of patients with a depressive episode, suicide attempters showed significantly higher levels of attachment anxiety but not attachment avoidance [27]. Kacmarski [28] also reported ambiguous direct associations between avoidant attachment and suicidality. Based on correlations between anxious attachment and loneliness, studies found a direct effect of anxious attachment on TB as well as a significant partial mediation of the effect on SI by TB for college students [28] and adolescent inpatients [29]. Contrary to expectation, avoidant attachment also showed a significant positive effect on TB. TB fully mediated the indirect effect of avoidant attachment on suicide risk [28]. These studies show that insecure attachment is related to belongingness needs and TB as well as suicide-related outcomes like SI. However, it remains unclear how anxious and avoidant attachment influence the strength of SI and if they also moderate the formation of SI from TB.

Due to the heterogenous finding on the relationship between TB and SI, the aim of the present study was to investigate the influence of the NTB and attachment on the appearance and intensity of SI with TB. Unexpectedly inconsistent or weak correlations can point to the moderating effect of another variable [30]. Therefore, we conducted moderation analyses to determine if the relationship between TB and SI differs due to a person’s attachment and NTB. Based on results about the NTB, relationship satisfaction, and SI (e.g., 12), we hypothesized that 1) the relation between TB and SI is significantly moderated by the NTB. In line with Leary et al. [11] and the conceptualization of avoidant attachment [19], we hypothesized that 2a) anxious attachment is positively correlated with the NTB, while 2b) attachment avoidance is negatively associated with the NTB. Therefore, we assumed that the effects of anxious and avoidant attachment neutralize each other and 2c) there is no association between the product of anxious and avoidant attachment and the NTB. Based on Kacmarski [28], we also hypothesized that 3a) anxious attachment and 3b) avoidant attachment both significantly moderate the relationship between TB and SI. The last hypothesis is, that 3c) the product of anxious and avoidant attachment is not a significant moderator of this relation. The possible covariates age and gender were investigated post hoc. We assumed no significant changes to the moderation analyses.

## Materials and methods

### Participants

The sample used for the analysis comprised *N* = 445 participants, aged 18–73 years (*M* = 29.90, *SD* = 11.64). Twenty-six people who spend less than 5 minutes filling out the questionnaires or failed the attention check item were excluded. Three hundred thirty-four (75.1%) participants were female. Of all participants, 135 (30.3%) had a mental disorder, 109 were currently undergoing therapeutic treatment, and 102 (22.9%) reported experiencing SI in the last week. Two hundred sixty-seven (60.0%) people were in a romantic relationship, 123 (27.6%) were currently single but had relationships in the past, and 55 (12.4%) never had a romantic relationship.

### Procedure

Participants were recruited between April and July 2021 through social media (Facebook, Instagram) and by contacting hospitals and psychotherapists in Germany. Participants had to be at least 18 years old and fluent in German. Data was collected anonymously using the online server www.soscisurvey.de. Participants were educated about the real purpose of the study and not misled. The announced aim of the study was to identify influencing factors on suicidal ideation and behaviour. Before filling out the survey, participants were also informed about the voluntary participation, anonymity, and data privacy. The study was approved by the ethic committee of the Department of Psychology, University of Duisburg-Essen, Germany, and is in accordance with the Declaration of Helsinki [31].

### Measures

The survey consisted of sociodemographic questions and a set of self-report questionnaires, including the following:

Need to Belong Scale (NTBS; 11, 32). The Need to Belong Scale includes ten items about the desire for acceptance and belonging (e.g., “I want other people to accept me.”), which must be answered on a Likert scale ranging from “1 = not at all” to “5 = completely”. Higher sum scores indicate a stronger NTB. Internal consistencies for the German version were good in this sample (Cronbach’s α = 0.82).

Bochum Adult Attachment Questionnaire (Bochumer Bindungsfragebogen, BoBi; 22). The German version of the Experience in Close Relationships (ECR; 21) was used to assess attachment in romantic relationships on the two dimensions anxious attachment (e.g., “I worry about being abandoned.“) and avoidant attachment (e.g., “I try to avoid getting too close to my partner.“) with 18 items each. All items do not refer to a specific relationship, but to general experience and behaviour in partnerships. A Likert scale from “1 = strongly disagree” to “7 = strongly agree“ was used. Internal consistencies for the avoidant (Cronbach’s α = .93) and anxious attachment scales (Cronbach’s α = .90) were excellent.

Interpersonal Needs Questionnaire (INQ; 5, German version: 33). The Interpersonal Needs Questionnaire measures PB (six items) and TB (nine items). All items are assessed on a Likert scale ranging from “1 = not at all true for me” to “7 = very true for me”. The present study only used the TB subscale (e.g., “These days, I feel disconnected from other people.“). Hallensleben et al. [33] reported very good internal consistencies for both scales. In the present sample, the TB subscale showed an excellent internal consistency (Cronbach’s α = .90) as well.

Beck Scale for Suicide Ideation (BSS; 34, German version; 35). Presence and strength of SI were measured with the Beck Scale for Suicide Ideation. Twenty-one groups of three statements are rated on a three-stage scale from “0” to “2”. Each item consists of different sentences representing low, moderate, and high intensity of SI. The total score is the mean of the first 19 items. Higher scores indicate stronger SI. Items 20 and 21 provide additional information on the frequency and seriousness of former suicide attempts. In line with Kliem et al. [36], the screening mean for SI, consisting of the mean score of items 1 to 5, was used for the analysis. Internal consistencies for the total score (Cronbach’s α = 0.89) and the screening score (Cronbach’s α = 0.92) were excellent in this sample.

### Statistical analysis

The program IBM SPSS Statistics 26.0 was used for all analyses. Participants who did not complete all questionnaires were excluded before the analyses. Due to the settings of soscisurvey.de, participants were not able to complete the questionnaires with skipping items which prevented missing data. Frequencies, mean values, standard deviations, and correlations were calculated to provide an overview of the descriptive statistics. Relations between attachment and the NTB (hypothesis 2) were calculated using bivariate correlation analysis and interpretated according to the guidelines of Cohen [37]. For hypotheses 1) and 3), moderated regression analyses were conducted using the PROCESS plug-in (version 3.5.3; 38). The requirements for a moderator analysis were checked (VIF < 10, tolerance > 0.1, Durbin-Watson-statistic = 1.98). A violation of the normal distribution and homoscedasticity was corrected by bootstrapping with 5000 iterations and a robust HC3 estimator of the standard error [39]. For the moderator analysis, variables were mean-centered [40], and effect sizes are reported with additionally explained variance as increments in R^2^ as well as with standardized beta coefficients [41]. Johnson-Neyman intervals and plots indicate at which levels of the moderator the moderation is significant [42]. A post hoc analysis was used to investigate age and gender as possible covariates. In the presentation of the results, the term prediction is used as a statistical wording. Nevertheless, it should be noted that data was only collected cross-sectionally.

## Results

### Descriptive statistics

Descriptive information of all questionnaires is displayed in Table 1. Sixty-one people (13.7%) stated that they attempted suicide once or more often in the past. A simple linear regression revealed that TB significantly predicted SI (*F*(443) = 236.69, *p* < .001).

**Table 1:** Descriptive statistics and correlations for sociodemographic information and all questionnaires

	m	w	1	2	3	4	5	6
1. Gender	24.7%	75.1%						
	*M*	*SD*						
2. Age	29.9	11.6	0.09					
3. Thwarted Belongingness	3.2	1.5	− 0.04	− 0.06				
4. Suicidal Ideation	0.2	0.4	− 0.02	0.06	0.59**			
5. Need to Belong	2.9	0.5	− 0.25**	− 0.16**	0.29**	0.24**		
6. Anxious Attachment	3.5	1.2	− 0.05	− 0.04	0.49**	0.37**	0.50**	
7. Avoidant Attachment	3.0	1.2	0.04	0.03	0.58**	0.47**	0.15**	0.32**

*Note. M* = mean, *SD* = standard deviation, ** significant on level < 0.001

### Moderation need to Belong (question 1)

**Fig. 2:**
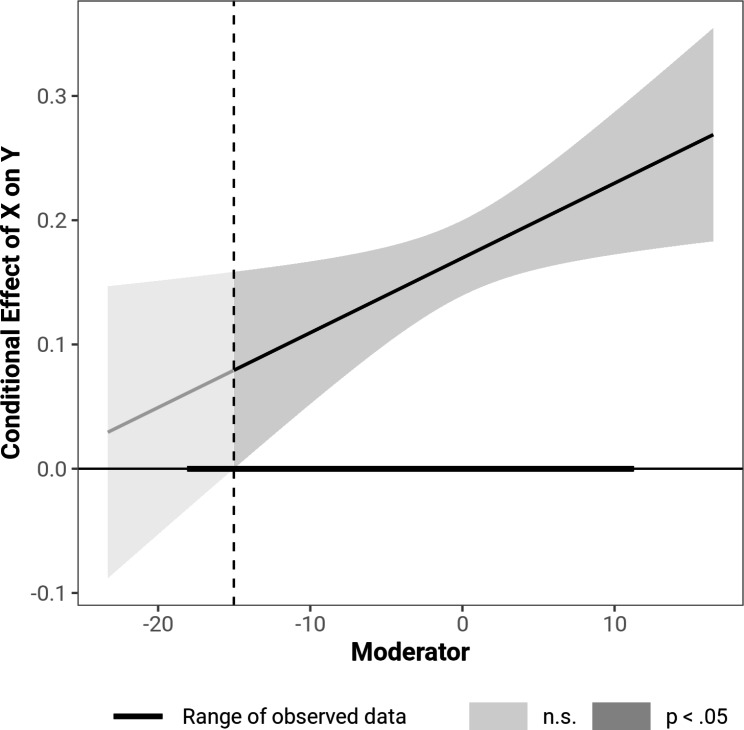
Johnson-Neyman plot indicating significance regions of the NTB as a moderator of the relationship between TB and SI

The moderation analysis revealed that the NTB moderated the relationship between TB and SI (β = 0.01, *t* (441) = 2.43, *p* = .01). The interaction term TB X NTB significantly predicted SI and explained 1.4% additional variance (Δ*R*^*2*^ = 0.014, *F* (1,441) = 5.90, *p* = .015). The relationship between TB and SI was stronger the higher the NTB was. Johnson-Neyman analysis showed that the NTB failed to moderate the association between TB and SI more than 2.7 SD below the mean, which only applied for the participant with the lowest NTB score with less than 13.89 of 50 points. This is displayed in the Johnson-Neyman plot in Fig. 2.

The post hoc analysis with age and gender as covariates revealed that age significantly influenced the moderation by the NTB (*p* = .03). Gender was not a significant covariate (*p* = .73). The significance of the moderation did not change from the inclusion of the covariates. Table 2 shows the complete results.

**Table 2:** Moderator analysis for NTB moderating the relationship between TB and SI with the covariates age and gender

Predictor	β	SE	*t*	*p*	Δ*R*^*2*^*(p)*
(Intercept)	0.11	0.07	1.70	0.089	
TB	0.17	0.02	11.11	0.000	
NTB	0.01	0.00	2.11	0.031	
TB x NTB	0.01	0.00	2.08	0.000	0.009 (0.039)
AGE	0.00	0.00	2.17	0.032	
GENDER	0.01	0.04	0.23	0.817	

*Notes.* TB = thwarted belongingness subscale of the interpersonal needs questionnaire, NTB = need to belong scale, TB x NTB = interaction between thwarted belongingness and the need to belong, β = standardized regression coefficient, SE = standard error, Δ*R*^*2*^ = additional variance explained

### Correlation need to Belong and attachment (question 2)

Anxious attachment was significantly associated with the NTB (*r* = .50, *p* < .001). This positive correlation is classified as strong [37]. Avoidant attachment correlated weakly positively (*r* = .15 *p* = .001) with the NTB. Moreover, the product of the anxious and avoidant attachment formed a new variable to include mutual effects of both attachment dimensions. The product aims for an operationalisation of mixed insecure attachment of both anxious and avoidant attachment at the same time, with lower values indicating more secure attachment. The product of both attachment dimensions was moderately associated with the NTB (*r* = .37, *p* < .001).

### Moderation attachment (question 3)

**Fig. 3:**
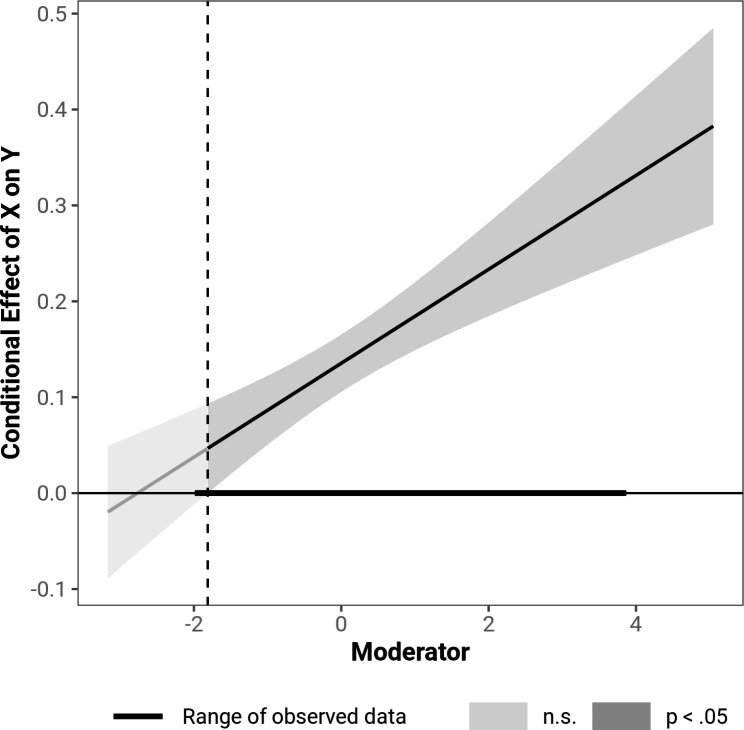
Johnson-Neyman plot indicating significance regions of avoidant attachment as a moderator of the relationship between TB and SI

The attachment dimensions as well as the product of anxious and avoidant attachment were tested as moderators of the relationship between TB and SI. All three moderator analyses were run separately. The analysis indicated a significant moderation of the relationship between TB and SI by anxious attachment (β = 0.04, *t* (3,441) = 3.14, *p* = .002). The interaction term of anxious attachment and TB explained 2.1% additional variance in SI (Δ*R*^*2*^ = 0.021, *F* (1,441) = 9.86, *p* < .001). Anxious attachment was a significant moderator at each level. However, the strength of the relationship between TB and SI increased with higher levels of anxious attachment. Avoidant attachment also moderated the relationship between TB and SI (β = 0.05, *t* (3,441) = 4.94, *p* < .001). The interaction explained an additional 4.0% of variance in SI (Δ*R*^*2*^ = 0.04, *F* (1,441) = 24.43, *p* < .001). As with anxious attachment, higher levels of avoidant attachment were associated with a stronger association between TB and SI. The Johnson-Neyman analysis showed that low scores of avoidant attachment 1.5 SD below the mean (1.14 points) no longer moderated the relationship between TB and SI, which applied to three participants. Figure 3 shows the Johnson-Neyman plot for avoidant attachment.

**Fig. 4:**
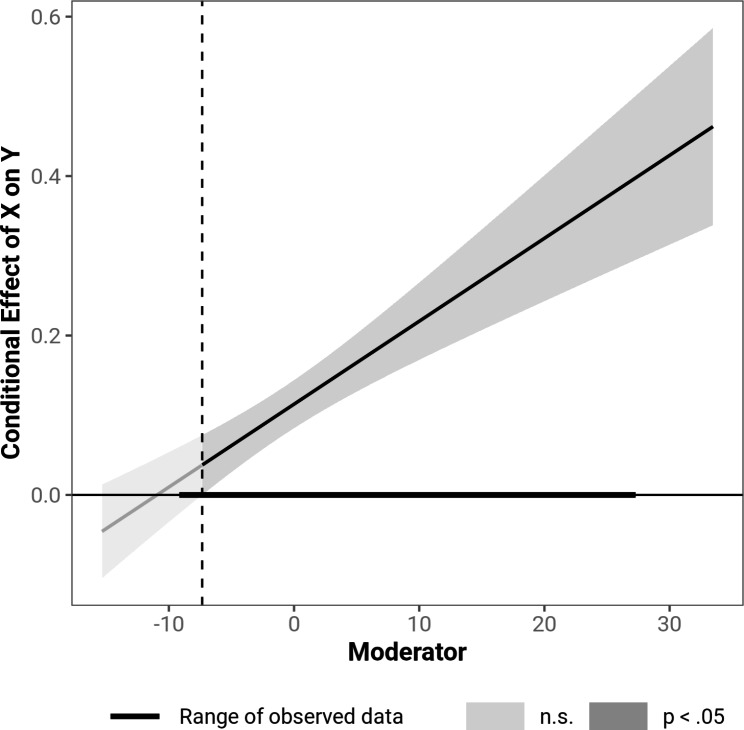
Johnson-Neyman plot indicating significance regions of the product of anxious and avoidant attachment as a moderator of the relationship between TB and SI

The results also indicate that the product of anxious and avoidant attachment moderated the relationship between TB and SI (β = 0.01, *t* (3,441) = 5.86, *p* < .001). 4.9% additional variance was explained (Δ*R*^*2*^ = 0.049, *F* (1,441) = 34.30, *p* < .001). A smaller product of anxious and avoidant attachment was accompanied by a weaker relationship between TB and SI. The moderation was no longer significant for values more than 1.2 SD below the mean, which applied to 7.2% of the participants. Figure 4 displays the area of a significant moderator as a Johnson-Neyman plot. All coefficients were not influenced by bootstrapping.

Age and gender were tested as covariates for each moderation analysis and did not reveal as significant covariates in the moderated regressions with attachment (all *p* > .05). The complete results are displayed in Table 3. All moderation analyses with attachment are still significant after covariates were included.

**Table 3:** Moderator analysis for attachment moderating the relationship between TB and SI

		β	SE	*t*	*p*	Δ*R*^*2*^*(p)*
Anxiety	(Intercept)	0.13	0.06	2.08	0.038	
	TB	0.16	0.02	8.79	0.000	
	Anxiety	0.04	0.02	2.00	0.042	
	TB x Anxiety	0.03	0.01	2.86	0.005	0.018 (0.005)
	AGE	0.00	0.00	1.90	0.058	
	GENDER	− 0.01	0.04	-0.20	0.843	
Avoidance	(Intercept)	0.11	0.06	1.81	0.072	
	TB	0.14	0.02	8.92	0.000	
	Avoidance	0.05	0.02	2.51	0.013	
	TB x Avoidance	0.05	0.01	4.79	0.000	0.037 (0.000)
	AGE	0.00	0.00	1.67	0.096	
	GENDER	0.00	0.04	0.11	0.916	
Anxiety X	(Intercept)	0.12	0.06	2.04	0.042	
Avoidance	TB	0.12	0.02	7.53	0.000	
	Anxiety x Avoidance	0.01	0.00	3.76	0.000	
	TB x Anxiety x Avoidance	0.01	0.00	5.58	0.000	0.045 (0.000)
	AGE	0.00	0.00	1.26	0.207	
	GENDER	− 0.00	0.04	0.02	0.982	

*Notes.* Anxiety = anxious attachment subscale of the Bochum adult attachment questionnaire, TB x Anxiety = interaction term of thwarted belongingness and anxious attachment, Avoidance = avoidant attachment subscale of the Bochum adult attachment questionnaire, TB x Avoidance = interaction term of thwarted belongingness and avoidant attachment, Anxiety x Avoidance = product of the anxious and avoidant attachment subscale indicating mixed insecure attachment, TB x Anxiety x Avoidance = interaction term of thwarted belongingness and the product of anxious and avoidant attachment, β = standardized regression coefficient, SE = standard error, Δ*R*^*2*^ = additional variance explained

## Discussion

Studies repeatedly reported heterogeneous results on the prediction of suicidal ideation (SI) by thwarted belongingness (TB) (e.g., 6). However, the conditions under which TB turns out as a significant predictor have not yet been clarified. The aim of the study was therefore to examine whether 1) the need to belong (NTB) significantly moderates the relationship between TB and SI and is 2) related to anxious and avoidance attachment such as with their product, and whether 3) the attachment dimensions are further moderators of the relationship between TB and SI. Consistent with Baumeister and Leary [10], all subjects reported having a NTB and differed in its intensity. The moderate correlation of the NTB with TB supports Van Orden et al. [4] who stated that TB, while being conceptually related to the NTB, is a distinct construct as a mental state arising when belongingness needs are not satisfied. In line with the Interpersonal Theory of Suicide [3], the strength of SI could be predicted by TB in this sample.

For hypothesis 1), the NTB was confirmed as a significant moderator of the relationship between TB and SI, with a stronger relationship at high levels of the NTB. This indicates that people with a high NTB are more vulnerable to SI when experiencing TB. This result aligns with Holden et al. [12] who found a similar effect on low relationship satisfaction and increased SI. Additionally, post hoc analyses revealed a significant influence of age on this effect. Negative correlations of age and the NTB suggest that younger people with a NTB could be especially vulnerable to develop SI from TB. In line with hypothesis 2a), the NTB was positively associated with anxious attachment, similar to Leary et al. [11]. Correlations of the NTB with avoidant attachment and the product of anxious and avoidant attachment, as mixed insecure attachment, were weaker but also positive. Hypotheses 2b) and 2c) were therefore not supported. Belongingness needs were higher in people with fear of being alone or abandoned but unexpectedly in people with motivation to prevent close relationships and dependency as well. The moderate positive correlation of insecure attachment with the NTB supports that both attachment dimensions go along with increased NTB. Finally, anxious and avoidant attachment significantly moderated the relationship between TB and SI, supporting Hypotheses 3a) and 3b). TB and SI were associated more strongly for people with higher levels of both anxious and avoidant attachment. While the direction of the effect for anxious attachment is consistent with the theory [21] and previous findings [28], the dimension avoidant attachment is described as leading to invulnerability to negative interpersonal feelings [19] and content without close relationships [21]. In contrast to that, our study unexpectedly showed that people with high avoidant attachment were more likely to develop SI from TB than people with low avoidant attachment. That is consistent with the correlations from hypothesis 2b) but previous studies regarding avoidant attachment and SI were ambiguous [28]. It is possible that people with high attachment avoidance repress their attachment needs by avoiding close relationships but are still subconsciously influenced by unsatisfied belongingness. Hypothesis 3c) was not confirmed because the product of anxious and avoidant attachment also moderated the relationship between TB and SI. Both anxious attachment and avoidant attachment seem to contribute to stronger development of SI when people experience TB.

### Practical implications

This study showed that a high NTB as well as insecure attachment can increase the likelihood of developing SI when belongingness needs are frustrated. Whether or not TB increases the probability and strength of SI can partially be explained by a person’s NTB and attachment security. Since SI is a key risk factor for suicide attempts [4], these findings have important practical implications for SI risk assessment and therapy. Diagnosis of SI, particularly for patients with depression or other mental disorders, should be extended beyond known predictors like TB and PB to the person’s NTB and attachment security. In line with the present results, elevated scores in the NTB and attachment insecurity can be indicators for a vulnerability to develop SI, especially when belongingness needs are not satisfied. It is important to consider both anxious and avoidant attachment patterns as well as the different associated behaviours, as the results show that avoidantly attached people avoid close relationships, even though they have high belongingness needs. The evaluation of a patient’s attachment experiences might also facilitate an adaptation of therapeutic goals and strategies. Anxiously and avoidantly attached individuals can be supported with different role-models and therapists encouraging desired strategies [23]. While attachment was not influenced by age and gender, a higher NTB was associated with younger age. Therefore, a patient’s NTB should be particularly addressed for young adults. Young adults could be more vulnerable to develop SI from TB because of higher belongingness needs as older adults. The results also offer an approach for prevention and treatment of SI. Patients with a high NTB or insecure attachment might particularly benefit from interventions to reduce loneliness and unfulfilled belongingness. Targeting TB for anxiously or avoidantly attached patients or people with a maladjusted NTB could effectively prevent the onset or lower the intensity of SI. The fact that loneliness and TB play a major role, especially in exceptional situations such as the contact restrictions during the COVID-19 pandemic [43], underlines their social relevance. Short et al. [44] reported promising results of computer-based intervention which could significantly reduce TB in a military sample and emphasized the potential of these low-threshold interventions with access from everywhere. A recent meta-analysis supported the effectiveness of interventions against loneliness and found that young people especially benefit from trainings promoting social and emotional skills [45]. Since the NTB is a relatively stable trait [10], Verhagen et al. [13] argued, that interventions to reduce the NTB are difficult and could produce negative side effects. Because a high NTB itself is not associated with decreased wellbeing [13] and SI [12], interventions should focus on the combination of a high NTB with loneliness and unfulfilled relationship satisfaction instead. Furthermore, the larger amount of explained variance from attachment insecurity in the present sample supports the promising impact on the prevention and treatment of SI. A study about attachment-based family therapy [46] suggests that working on attachment security can have direct effects on SI in adolescents. Participants who underwent attachment-based family therapy showed significantly more improvement in suicidal symptoms compared to standard therapy. Through more differentiated insights into attachment styles and associated behaviours, interventions could be better adapted to the experiences of insecurely attached individuals. This could be especially important for patients with borderline personality disorder who are particularly vulnerable to severe insecure attachment [47]. Future studies should therefore reassess these factors in a corresponding sample. This illustrates that attachment insecurity offers a therapeutic starting point with great potential that should be further explored.

### Strengths and limitations

This study has several strengths and limitations. There have been some studies examining attachment [28] which found mediated effects over TB on SI. However, the examination of a moderation of these factors in the present study brings new insights. Additionally, this is the first study examining NTB and attachment security in an adult sample, since many existing studies on NTB and attachment security have been conducted with adolescents (e.g., 13, 24). One limitation is that the answers in self-report questionnaires could be skewed by biases such as socially desirable responses [48]. However, an objective assessment of the investigated constructs is difficult. The data collection in a cross-sectional design should be validated in longitudinal studies to investigate causal relationships. Although women and students were overrepresented in this study, the sample was quite large with over 400 participants with heterogeneous occupations and demographics. Due to anonymous data collection, we could only assess self-reported diagnoses. However, in this sample 30.6% of the participants reported a mental disorder. That is similar to the prevalence of mental disorders in Germany between 27.8% for the general population and 36.7% for young people between 18 and 34 years [49]. In this regard, our sample can be seen as a representation of the German general population. This makes it easier to transfer results in clinical practice. This is why we did not include diagnosis as a covariate.

Anxious and avoidant attachment are conceptualized as unrelated dimensions and measured in separate scales [22]. However, other studies (e.g., 28) found a significant correlation of anxious and avoidant attachment. In our sample, anxious and avoidant attachment were only weakly related. The product of anxious and avoidant attachment in hypotheses 2c) and 3c) aims to still include mutual effects of the different attachment dimensions on the NTB, TB, and SI. Future studies with stronger correlations of the attachment dimensions should consider a rotated solution. For the interpretation, a possible influence of the COVID-19 pandemic and associated restrictions before and during the data collection in April to July 2021 should be considered. In our sample, 57.8% of the participants stated that their personal life was very or very heavily burdened by the pandemic. Even though overall suicide risk remained stable [50], recent studies support social consequences of the pandemic. Gratz et al. [51] found that TB increased due to social distancing and mediated the indirect effect of stay-at-home rules on suicide risk.

### Conclusion

The aim of the present study was to explore new approaches for the prediction of SI by TB. Attachment and the NTB turned out to be significant moderators of the relationship between TB and SI. It was surprising that not just anxious attachment, but also avoidant attachment increased the probability of SI. Hence, anxious, and avoidant attachment patterns as well as a high NTB are risk factors for the development of SI in people with TB. Therefore, these constructs should be included in the diagnosis and prevention of SI and could offer helpful approaches for therapy. Future studies should explore these effects for alternative conceptualizations of attachment and with clinically relevant samples like patients with borderline personality disorder.

## Data Availability

Data is available upon reasonable request from the corresponding author.

## Abbreviations

TB: Thwarted Belongingness
PB: Perceived Burdensomeness
SI: Suicidal Ideation
NTB: Need to Belong
